# Effect of Contrasting Redox Potential Evolutions and Cap Management Techniques on the Chemical Composition of Red Wine

**DOI:** 10.3390/molecules30153172

**Published:** 2025-07-29

**Authors:** Dallas J. Parnigoni, Sean T. Kuster, Jesus Villalobos, James Nelson, Robert E. Coleman, L. Federico Casassa

**Affiliations:** 1 Wine and Viticulture Department, California Polytechnic State University San Luis Obispo (Cal Poly), San Luis Obispo, CA 93407, USA; dparnigo@calpoly.edu (D.J.P.); skuster@calpoly.edu (S.T.K.);; 2 Department of Electrical and Computer Engineering, University of California, Davis, CA 95616, USA; jjnel@ucdavis.edu; 3 MeshVines, Davis, CA 95618, USA; 4 Department of Viticulture and Enology, Washington State University, Richland, WA 99354, USA; robert.coleman@wsu.edu

**Keywords:** cap management, automation, oxidation-reduction potential, glutathione, phenolic

## Abstract

This study investigated the effects of six cap management protocols targeting contrasting oxidation-reduction potential (ORP) evolutions during alcoholic fermentation of Pinot noir wines. Treatments included twice-daily punch-downs (PD) and pump-overs (PO), 1 h air or N_2_ injections (AirMix, N_2_Mix), air injections triggered by ORP ≤ −40 mV (RedoxConAir), and equal N_2_ injections concurrent to RedoxConAir wines (RedoxConN_2_). AirMix wines maintained ORP values above 0 mV throughout fermentation, showed an oxidatively favored glutathione-to-glutathione disulfide ratio (GSH:GSSG) of 0.3:1, and had 21% lower total phenolics and 24% lower anthocyanins than PD wines. In contrast, N_2_Mix wines maintained the lowest ORP, near −100 mV, and showed a reductively favored GSH:GSSG ratio (7:1). PD wines extracted 48% more flavan-3-ols than PO wines, consistent with greater berry integrity disruption and seed submersion. Volatile composition was also impacted: ethyl n-octanoate showed the highest OAV among esters, ranging from 147 in PO wines to 116 in AirMix wines. Results suggest the GSH:GSSG ratio served as an indicator of redox history, with potential implications for color and aroma preservation during aging. Inert gas mixings resulted in equal or greater total phenolic content, while excessive air injections may provide a tool to soften astringency.

## 1. Introduction

Pinot noir, believed to have originated in Burgundy, France, has been cultivated since at least the 14th century [1]. This varietal has demonstrated adaptability to many distinguished winegrowing regions globally and is the second most planted red wine grape variety in California [2,3]. Pinot noir grapes are generally low in most phenolic compounds, producing wines with lighter color and lower astringency. However, Pinot noir often contains high concentrations of the bitter phenolic compounds known as flavan-3-ols, including (+)-**catechin**, (−)-**epicatechin**, epigallocatechin, and **epicatechin gallate** [4,5,6]. Tailoring cap management protocols to facilitate the selective extraction of desired phenolics is of key importance in varietals such as Pinot noir, which may be particularly sensitive to winemaking conditions, when compared to other varietals [7,8].

During alcoholic fermentations of red wine, the release of carbon dioxide by yeast results in the rising of grape skins, seeds, and other solids upward, forming a cap near the surface of the fermenting juice. In the absence of mixing, this cap typically remains about two-thirds submerged below the liquid surface during alcoholic fermentation. Various winemaking interventions know as cap management are utilized to increase the contact of the solids with the fermenting juice, homogenize chemical and temperature gradients through physical mixing, provide oxygen to the must, and in more recent work, modulate the oxidation-reduction potential (ORP) [9]. While a variety of cap management techniques are available to winemakers [7,10,11,12,13,14], among the most common industry practices are punch-downs and pump-overs. Regarding these two protocols, few direct comparisons exist in the literature with results potentially depending on the length of time that these operations are performed and further variability resulting from the type of pump-over employed [12].

Cap management techniques are typically implemented 1 to 3 times per day during alcoholic fermentation, with their frequency reportedly affecting the sensory composition of the resulting wines [15,16]. Punch-downs are executed using a plunger to physically push the cap into the fermenting juice, achieving physical mixing and increasing juice to skin contact. This practice disrupts berry integrity and has been shown to increase anthocyanin, tannin, and polymeric pigment concentrations when compared to simply submerging the cap throughout alcoholic fermentation [14]. However, previous research has shown only minor differences in tannin and total phenolic content between wines made with no cap management and those made with up to three punch-downs daily [15]. Punch-downs are thought to supply the juice with a relatively small amount of oxygen, while alternative cap management practices such as pump-overs can be implemented with varying levels of oxygen incorporation [17].

Pump-overs employ a pump to transfer fermenting wine from the bottom portion of the vessel to the top, wetting the surface of the cap. Typically, a total of one-half to two times the volume of the wine is pumped-over in each cap management session, with variable effects related to frequency and volume transferred [18]. Unlike punch-downs, pump-overs do not completely break apart or submerge much of the grape solids in the cap. As a result, the cap largely remains intact and at the surface, especially in large volume fermentors. Additionally, both temperature and chemical gradients may persist even after energic pump-overs. For example, one study reported a 14 °C temperature differential between the cap relative to the juice just 3.3 h after pump-over in 3450 L Pinot noir fermentations [19]. Similarly, gradients of skin phenolics in 2000 L fermentation of Cabernet Sauvignon reached saturation limits near the cap eight hours after pump-overs [16]. Lastly, previous research reported open-to-air pump-overs performed with venturi injectors allowed for the dissolution of 3 mg O_2_/L per cap management session. However, approximately 80% of the dissolved oxygen was confined to the upper portion of the tank [17], an issue that may be resolved by direct air injections to the must.

In addition to supplying yeast with oxygen and positively impacting fermentation rate and cell biomass [20,21], air injections also influence the oxidation-reduction potential (ORP) [22,23,24,25]. The ORP reflects the tendency of redox-active species in solution to accept (reduction) or donate (oxidation) electrons, indicating the overall oxidative or reductive state of the system. Unlike dissolved oxygen (DO), which is rapidly consumed by yeast and displaced by carbon dioxide (CO_2_), ORP describes in real-time the oxidative or reductive state of the wine in the anaerobic or semi-aerobic conditions of alcoholic fermentations [9,26]. This was highlighted in a previous study, which noted exceedingly low DO concentrations despite constant air sparging rates of 200 mL_air_/L_liquid_/min^−1^ in fermentations of very-high-gravity model solutions. In the same study, ORP was measured and controlled at varying minimum potentials with air injections, despite near 0 DO concentrations [27].

Similarly, Nelson et al. [24] maintained a −40 mV ORP minimum during alcoholic fermentation of wine both at research and commercial scale. This setpoint was selected as the potential at which 99.9% of the redox couple sulfur (S) and hydrogen sulfide (H_2_S) is likely to exist in the non-volatile elemental sulfur state, effectively preventing the accumulation of malodorous H_2_S that may otherwise occur due to chemical reduction [24].

Importantly, accurate interpretation of ORP values requires consideration of the electrode used, as well as the dominate redox active species in a given solution. Several types of reference electrodes are available, each optimized for specific media and applications, including cyclic voltammetry and the Briggs–Rauscher method for the determination of antioxidant activity [28,29,30]. The wine industry has largely employed the silver/silver chloride (Ag/AgCl) electrode with a 3 M potassium chloride (KCl) salt bridge. This probe design produces a stable voltage of approximately 220 mV; therefore, 220 mV is added to the provided values to relate to the standard hydrogen electrode (SHE). While some research has reported values relative to the SHE regardless of the probe used, many instead report the value provided by the given reference electrode. Due to this incongruous reporting of ORP values, these electrode-specific offsets become important when comparing ORP data across studies. In the present study, all values reported and referenced are related to the Ag/AgCl reference electrode.

During alcoholic fermentation, the redox-active species that dominate the ORP in wine are primarily limited to the Fe^2+^/Fe^3+^ and Cu^+^/Cu^2+^ couples, the glutathione and glutathione disulfide couple (GSH/GSSG), and H_2_O_2_ formed from O_2_ through Fenton reactions [9,31,32]. Tartrate complexes with transition metals influence the propensity of these metals to participate in redox reactions [33,34], effectively influencing the redox buffer capacity of wine, in tandem with phenolic compounds [35]. During alcoholic fermentation, air additions were shown to increase the ORP by up to 300 mV [8,23], followed by a rapid decline that is thought to be mediated by reductive species including glutathione (GSH) and the regeneration of Fe^2+^ from Fe^3+^ [9,33].

GSH is a ubiquitous tripeptide antioxidant in biology and is present in animal, plant, and fungi cells, including grapes, yeast, and humans. In yeast, GSH is synthesized both to regulate the intracellular redox potential and to influence the extracellular local environment by exporting GSH [36,37]. The ratio of GSH to GSSG has been proposed as a measure of oxidative stress in both microbiology [38] and medicine [39], a concept that is equally relevant in winemaking as a marker of a wine’s antioxidant potential and oxidative history. Due to these antioxidant capabilities, GSH additions are sometimes applied during winemaking with the goal of preserving color and aroma. For example, in sparkling wines supplemented with 20 mg/L GSH before bottling, reduced browning was observed after 12 months of bottle aging [40], and reduced yellowing and increased retention of varietal aromas was observed in Sauvignon blanc wines [41]. Additionally, previous work detailed the preservation of esters in white wines with reduced SO_2_ when supplemented with 10 mg/L glutathione after alcoholic fermentation [42].

While the impact of cap management on wine’s phenolic and volatile composition has been widely researched [8,12,14,43], studies concerning the interactions between cap management, ORP evolution during alcoholic fermentation, glutathione chemistry, and the subsequent phenolic and volatile chemistry of red wines remain scarce. Additionally, to the best of our knowledge, the use of air and inert gas injections to automate cap management while monitoring ORP during alcoholic fermentation has not previously been investigated.

This study extends previous research that explored phenolic, volatile, and sensory differences among red wines produced using various cap management approaches, including punch-downs, pump-overs, no cap management, and 1 h air or N_2_ gas mixings [8]. Building on these findings, the current work investigates similar treatments: punch-downs (PD), pump-overs (PO), 1 h air mixing (AirMix), and N_2_ mixing (N_2_Mix). In addition to the original treatments, an ORP dictated treatment was added whereby air was injected into the fermenting must if the ORP declined to −40 mV (RedoxConAir). A parallel treatment injected N_2_ into the must if the corresponding replicates of RedoxConAir wines received an air injection (RedoxConN_2_). The objective of this study was to investigate how cap management protocols designed to produce contrasting ORP evolutions during alcoholic fermentation may alter the phenolic profile, volatile composition, and glutathione-related chemistry of red wine.

## 2. Results and Discussion

### 2.1. Alcoholic Fermentation of the Wines

**Figure 1:**
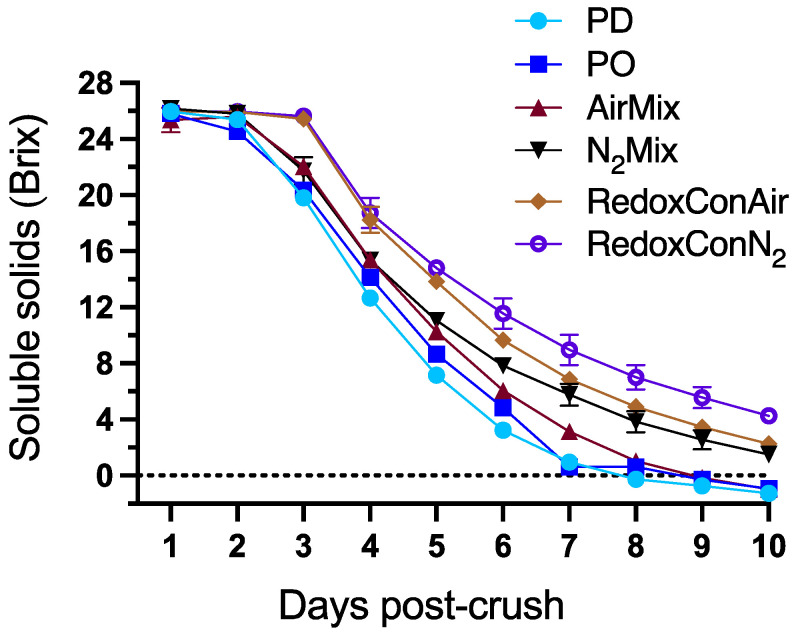
Evolution of sugar consumption measured as Brix during alcoholic fermentation of Pinot noir wines produced with selected cap management protocols. Each data point represents the mean of three replicates (*n* = 3) with error bars representing the standard error of the mean.

Fermentation kinetics of sugar consumption and temperature evolutions of the wines are shown in Figure 1 and Figure S1, respectively.

An extended lag phase observed in all Pinot noir wines, lasting about two days, indicated a sluggish alcoholic fermentation typically associated with low yeast biomass [44]. Previous research has detailed the importance of oxygen in the cell growth phase of alcoholic fermentation due to its role in sterol and unsaturated fatty acid synthesis by *Saccharomyces cerevisiae*, which significantly influences the ethanol tolerance, fermentative capability, and viability of yeast [45].

While the programmable logic controller was set to inject air into RedoxConAir wines if the ORP dropped to −40 mV or below, this limit had not been reached in the first 2 days of maceration, due to the absence of active fermentation. In turn, these wines received no cap management or air injection until a manually activated 1 h air mixing was applied approximately 40 h after yeast inoculation. Similarly, N_2_Mix and RedoxConN_2_ wines received no supplementary air throughout alcoholic fermentation. Consequently, N_2_Mix, RedoxConAir, and RedoxConN_2_ wines failed to reach 0 Brix during the 10-day maceration. Previous research has shown successful completion of alcoholic fermentation within as few as four days in cases of no cap management or nitrogen mixing, and therefore no oxygen additions [8]. The present results suggest that under conditions of potentially low yeast biomass and cell viability, as indicated by the extended lag phase and sluggish fermentation, oxygen additions may be necessary to ensure the onset and progress of alcoholic fermentation.

Although N_2_Mix, RedoxConAir, and RedoxConN_2_ wines each contained >20 g/L of glucose and fructose at pressing, these wines continued to consume sugars and were ultimately bottled with <5 g/L glucose and fructose and were considered effectively dry.

### 2.2. The Oxidation-Reduction Potential During Alcoholic Fermentation

**Figure 2:**
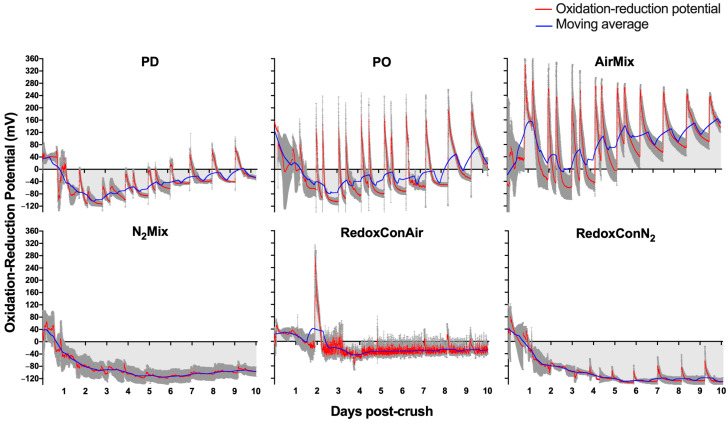
Oxidation-reduction potential (ORP) evolutions of Pinot noir wines during alcoholic fermentation performed with selected cap management protocols. Gray shading represents the standard error of the mean (*n* = 3). ORP values are reported versus an Ag/AgCl reference electrode.

Figure 2 illustrates the contrasting ORP evolutions observed during the course of the various cap management protocols. PD and PO wines contrasted in the magnitude of ORP increases during cap management, with typical increases of 60 to 80 mV and 160 to 180 mV due to punch-downs and pump-overs, respectively. However, the moving average of ORP across alcoholic fermentation remained similar between these two treatments until day 8, after which PO wines showed a more pronounced upward trend above 0 mV, while PD wines remained largely negative albeit with an upward trend as well. The reduced yeast activity and subsequently reduced GSH production and oxygen consumption at the end of alcoholic fermentation likely influenced this upward trend, suggesting that at this stage pump-overs may have a more lasting oxidative effect on the ORP than pump-overs applied during peak alcoholic fermentation (days 2, 3). Additionally, both PD and PO wines reached minimum ORP values of near −100 mV during days 2 and 3 of alcoholic fermentation.

AirMix wines showed a greater oxidative response to cap management than all other treatments. In response to the 1 h air injections, peak ORP values often exceeded 250 mV, roughly 300 mV above typical ORP values before cap management during the peak of alcoholic fermentation (days 2, 3). These wines were the only wines of all treatments that maintained moving average ORP values above 0 mV throughout alcoholic fermentation, explained by the approximately 2940 L of air cumulatively injected to each replicate during the 10-day alcoholic fermentation.

In contrast, N_2_Mix and RedoxConN_2_ wines declined in ORP following inoculation of *Saccharomyces cerevisiae* and equilibrated with minimal response in ORP to injections of inert N_2_ gas. Minimum ORP values of −115 mV were recorded in these treatments, and unlike PD, PO, and AirMix wines, the N_2_ based treatments did not result in an upward trend in the moving average ORP over time.

Lastly, RedoxConAir wines responded to 10-second air injections with 20 to 40 mV increases, successfully maintaining a minimum ORP of −40 mV. A single 1 h air injection was applied on day 2 of maceration to encourage the start of alcoholic fermentation. Other than the 210 L of air injected in this single event, the −40 mV setpoint was maintained with a total of approximately 110 L of air across the entirety of the 10-day alcoholic fermentation. This amounted to slightly less than one 10-second air injection per hour on average during the duration of fermentation. Considering that the air was delivered through 3.2 mm diameter tubing, these were not highly nebulized bubbles, and the oxygen dissolution was likely quite minute in relation. This shows the sensitivity of the ORP, and the probes used, to even relatively small oxidative events.

In summary, the contrasting cap management protocols exerted distinct effects on the ORP evolution of the wines during alcoholic fermentation. Although PD and PO wines exhibited similar moving averages, the oxidative influence of open pump-overs produced relatively brief spikes in ORP to near or above 160 mV. AirMix wines maintained positive ORP values throughout most of alcoholic fermentation, showing the magnitude of oxidative effect direct air injections may have, even when compared to two-volume, open system pump-overs. Conversely inert N_2_ gas injections to N_2_Mix and RedoxConN_2_ wines did not affect the ORP; thus, these wines maintained consistently low ORP values near a −100 mV baseline throughout alcoholic fermentation. Lastly, RedoxConAir wines were limited to the minimum ORP of −40 mV, effectively maintaining an ORP environment unlikely to facilitate significant chemical reduction of elemental sulfur to H_2_S, thereby reducing the risk of this wine fault. Importantly, this was achieved using a relatively small volume of air, injected through 3.2 mm inside diameter plastic tubing. This result suggests that relatively large-bubble air delivery through low-cost tubing may offer a practical alternative to fine-pore sinter elements, which are typically more specialized and costly to implement.

Given the observed modulation of ORP across treatments, further investigation into redox-active compounds, particularly glutathione and its derivatives, was carried out to better understand their roles in shaping the oxidative or reductive environment during fermentation.

### 2.3. Glutathione and Glutathione Derivative Composition of the Wines

**Figure 3:**
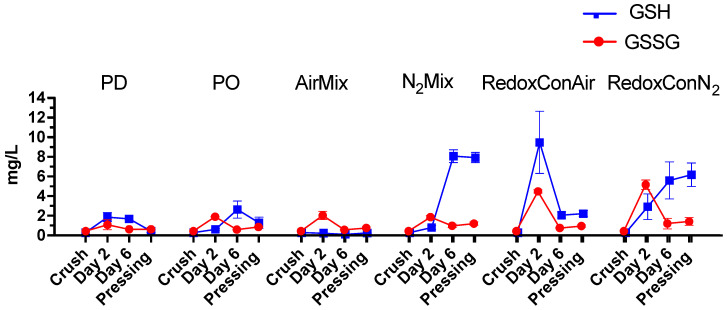
Glutathione (GSH) and glutathione disulfide (GSSG) concentrations during alcoholic fermentation of selected cap management protocols in Pinot noir wines. Each data point represents the mean of three replicates (*n* = 3) with error bars representing the standard error of the mean.

**Figure 4:**
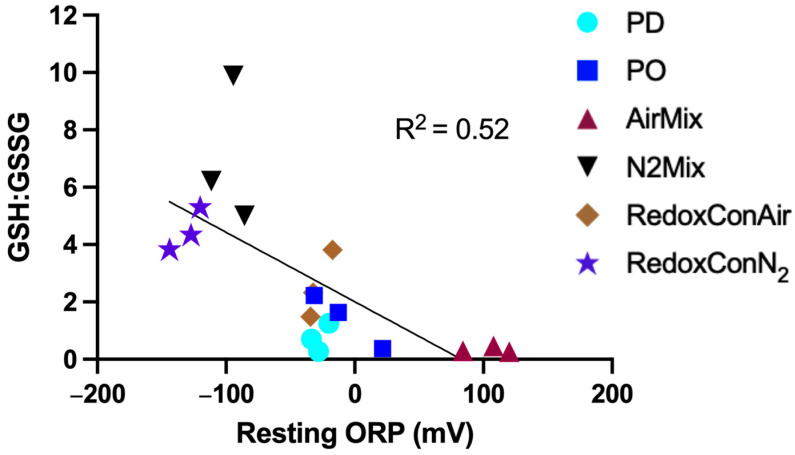
Linear regression of the GSH:GSSG ratio and the resting ORP at the end of alcoholic fermentation (i.e., pressing) of Pinot noir wines made with selected cap management protocols.

Glutathione (GSH) is present in grapes and is actively produced by yeast during alcoholic fermentation of wine [46]. It plays a key role in modulating both intra and extracellular ORP of the yeast [37,47]. In grape must, GSH functions as a scavenger of quinones, including the quinone of caftaric acid, leading to the formation of 2-S-glutathionyl caffeoyl tartrate, or grape reaction product (GRP) [48,49,50]. Alternatively, GSH may regenerate quinones back to their corresponding phenols, and itself oxidizing to produce glutathione disulfide (GSSG) [48]. It is therefore of interest to investigate the concentrations of the GSH/GSSG couple as well as the glutathione derivative GRP formed during alcoholic fermentation. Herein, the evolution of these compounds during winemaking and aging was followed (Figure 3), and the relationship between glutathione derivatives and the ORP is explored (Figure 4).

Initial measurements of GSH and GSSG taken immediately after crushing revealed relatively low concentrations of glutathione present in the juice (0.3 mg/L GSH; 0.4 mg/L GSSG). However, these compounds accumulated uniquely between treatments during alcoholic fermentation. On day 6 and at pressing, N_2_Mix and RedoxConN_2_ wines contained significantly more GSH than all other treatments. Notably, the ratios of GSH:GSSG at pressing also strongly favored the reduced state of the redox couple in N_2_Mix (7:1) and RedoxConN_2_ wines (4.4:1), which likely contributed to the low ORP at the end of alcoholic fermentation in N_2_Mix and RedoxConN_2_ wines [47,51].

In contrast, PD wines showed GSH:GSSG ratios of 0.7:1, that is, shifted to the oxidative state despite these wines experiencing relatively modest oxidative events during alcoholic fermentation, according to the ORP evolution (Figure 2). Interestingly, PO wines appeared to produce more GSH during alcoholic fermentation than PD wines and resulted in a GSH:GSSG ratio of 1.4:1 at pressing. This was shifted to the reduced state relative to PD wines. It is hypothesized that the short-term oxidative spikes during pump-overs may have spurred an increased GSH production by yeast responding to oxidative stress [52].

AirMix wines exhibited the lowest concentration of GSH throughout alcoholic fermentation consistent with higher oxygen exposure and an elevated ORP trend. It is likely that any increased GSH production by yeast was overwhelmed by the quantity of hydroxycinnamic acid quinones produced during air injections. Accordingly, the observed GSH:GSSG ratio of AirMix wines at pressing was of 0.7:1 and thus shifted toward the oxidized state (Figure 3).

A regression analysis of the ratio of GSH:GSSG and the average ORP in the hour prior to pressing revealed a moderate relationship between these two variables (*p* = 0.04; R^2^ = 0.52) (Figure 4). We hereby define “resting ORP” as “the ORP measured after the short term, transient effects of cap management have dissipated, and the system has returned to a more stable redox state”. In the present study, the resting ORP was best represented by the average ORP during the hour prior to pressing. Figure 4 suggests that glutathione may influence the resting ORP at the end of alcoholic fermentation. Therefore, the GSH:GSSG ratio may serve as a marker for the oxidative or reductive history of wine.

While the correlation is significant, model fitness may be improved by using ORP and GSH:GSSG values obtained after a more complete ORP stabilization, potentially several days post-pressing. In the present study, PO and AirMix wines were still declining from cap management approximately 24 h prior (Figure 2), while RedoxConAir wines were actively maintained at −40 mV, preventing natural ORP equilibration. As a result, the observed ORP values may not fully reflect the corresponding glutathione redox state. Future studies should consider allowing ORP to stabilize before assessing glutathione composition to better capture this relationship.

**Figure 5:**
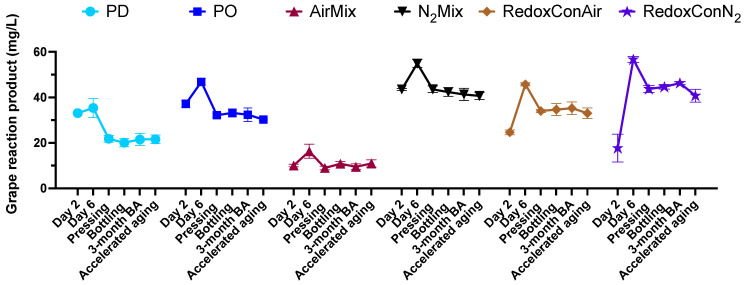
Concentration of the grape reaction product (GRP) at selected time points during winemaking, bottle aging (BA), and accelerated aging of Pinot noir wines produced with selected cap management protocols. Points represent the mean of three tank replicates (*n* = 3), and bars represent the standard error of the mean.

GSH has been shown to form adducts with the quinones of various polyphenols in red wine, including flavan-3-ols and other hydroxycinnamic acids, or regenerate their corresponding catechol via redox reactions [29]. GRP formation and retention may serve as an indicator of the total glutathione–quinone adduct concentration of the wines, displaying the antioxidant activity of GSH during alcoholic fermentation. GRP concentrations at pressing were found to be related to the ORP evolutions and cap management protocols of the wines (Figure 5).

N_2_Mix and RedoxConN_2_ wines showed the highest concentration of GRP at pressing, followed by PO and RedoxConAir wines. The similar decrease in GRP as was seen in decreases of GSH in PD wines further supports the hypothesis that the slightly oxidative conditions of PO and RedoxConAir wines encouraged the production of GSH by yeast, ultimately resulting in a greater binding of quinones and thus an increase in GRP, when compared to PD wines.

AirMix wines contained the least GRP, with just 20% of the concentration measured in N_2_Mix and RedoxConN_2_ wines at pressing. It is likely that the prolonged aerations of AirMix wines led to the formation of quinones in excess of the available GSH, resulting in the oxidation of GRP and further polymerization reactions with phenolic compounds or the formation of 2,5-di-s-glutathionyl caffeoyl tartaric acid (GRP2) [48,49,53]. However, GRP2 was not quantified in the present study.

Overall, PD wines showed lower GSH production during fermentation compared to PO and RedoxConAir wines, despite showing a similarly reductive ORP evolution. In contrast, N_2_Mix and RedoxConN_2_ wines consistently showed reductive environments during alcoholic fermentation, likely preserving GSH and stabilizing GRP by minimizing oxygen exposure and subsequent quinone formation [48,51]. This retention of GSH may offer extended antioxidant protection into wine aging [54]. AirMix wines, subjected to frequent oxidative events, exhibited the lowest GRP concentrations, suggesting further oxidation of GRP to downstream products. Previous research has shown GRP to be a substrate for oxidation by laccase but not by other polyphenol oxidases; however, visual inspection of the grapes of the present study confirmed negligible laccase containing *Botrytis cinerea*, suggesting further research may be needed to confirm the mechanisms for the GRP losses in AirMix wines [49,53,55]. These results highlight the relationship among cap management, ORP dynamics, and glutathione composition and suggest that the GSH:GSSG ratio may serve as a meaningful indicator of the oxidative history of a given wine. Therefore, the present results indicate that choice of cap management applied may in turn affect aging potential of the wine mediated by the antioxidant action of GSH.

### 2.4. Basic Chemistry of the Wines

Relatively minor differences were found in ethanol, pH, titratable acidity (TA), malic, lactic, and acetic acid concentrations among the wines of all treatments in the present study (Table S1). Alternatively, the study that this research builds upon reported roughly double the acetic acid in Petite Sirah wines produced with 1 h air injections when compared to PD wines [8]. However, the previous study delivered air with 2 µm pore size sinter elements rather than 3.2 mm tubing as in the present study, likely leading to a greater rate of oxygen dissolution. Additionally, all AirMix wines of the previous study completed alcoholic fermentation by day 6, but air injections continued until day 9. AirMix wines of the present study did not reach 0 Brix until day 9, at which point air injections were ceased. Negative Brix is anticipated at the end of alcoholic fermentation, and occurs soluble solids, i.e., sugars, reach low levels, while ethanol, being less dense than water, accumulates, resulting in negative values when using a densitometer. The continued yeast activity during the entirety of the cap management period likely provided a continued anaerobic environment not suitable to acetic acid bacteria in the present study. This result emphasized wine’s sensitivity to air injections after the completion of alcoholic fermentation. This result also highlighted the potentially desirable effects on tannin softening and phenolic development that may be achieved without the risk of acetic acid accumulation if air mixing is applied exclusively during alcoholic fermentation.

### 2.5. Phenolic Composition of the Wines

**Figure 6:**
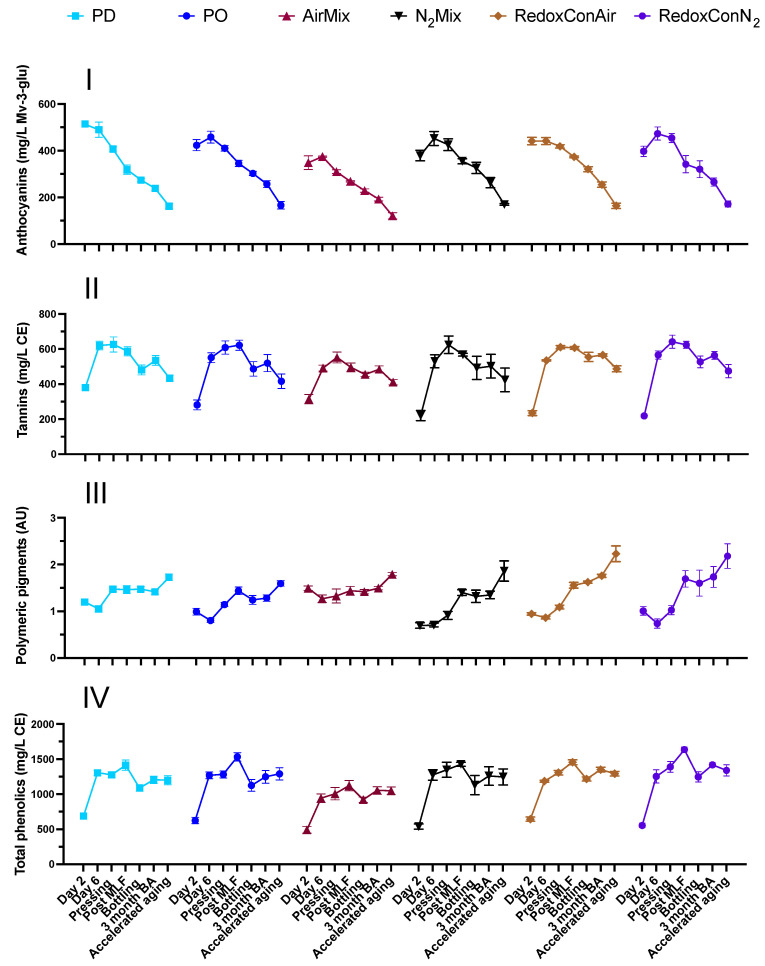
Phenolic measurements of (from top to bottom) anthocyanins (**I**), tannins (**II**), polymeric pigments (**III**), and total phenolics (**IV**) at selected stages of winemaking, bottle aging (BA), and accelerated aging. Points represent the mean of three tank replicates (*n* = 3), and bars represent the standard error of the mean.

The phenolic composition of wine largely dictates the chromatic (i.e., visual) and astringent (i.e., mouthfeel-tactile) qualities of wine and contributes to its antioxidant capacity as well. Many of these compounds are largely extracted into juice or wine during maceration of grape solids. While volatile phenols may be present in wine and affect wine aroma, the present study focused on the nonvolatile phenolic compounds described herein. Given that a key goal behind the application of all cap management practices is to facilitate the extraction of phenolic compounds, it is crucial to investigate the unique outcomes of contrasting cap management protocols on said extraction. Anthocyanin, tannin, polymeric pigment, and total phenolic concentrations were measured during winemaking, aging, and accelerated aging (Figure 6, Table S2).

At pressing, wines of all treatments other than AirMix contained comparable concentrations of anthocyanins, tannins, and total phenolics, while AirMix wines showed lower concentrations of anthocyanins and total phenolics. The comparable or increased phenolic extraction and color in wines produced without punch-downs or pump-overs, such as through N_2_ mixing or no cap management has been previously shown [8]. Phenolic extraction in the absence of physical cap disruptions and/or cap management was previously attributed to pressure on the cap resulting from the net forces of atmospheric pressure, and the upward pressure of CO_2_ produced by yeast in vessels of favorable size, geometry, and temperature [56]. While these net forces may compress the cap and facilitate extraction, minimal wetting or submerging of the cap (as occurs during punch-downs and pump-overs) may additionally result in the accumulation of heat in the cap [57], further facilitating extraction from grape solids.

The most distinct modulation of phenolic chemistry was observed in AirMix wines, consistent with previous findings [8]. At pressing, AirMix wines had 24% less anthocyanin than PD wines, as supported by full visible spectrum scans (Figure S2). However, differences in anthocyanins between treatments lessened with age, ultimately resulting in only negligible differences in anthocyanins between treatments after accelerated aging (Table S2). Tannin concentrations followed a similar pattern, with AirMix wines showing a 12% decrease when compared to PD wines at pressing; however, due to substantial standard deviations, this result was not statistically significant.

Reductions in phenolic concentrations observed in AirMix wines could be partially ascribed to the activity of polyphenol oxidases (PPO). A previous study examined the contrasting oxidation rates of chemical oxidation versus laccase-mediated enzymatic oxidation of various phenolic compounds [58]. In that study, chemical oxidation of catechol and phenol were reported to occur at <5 and <1 μmol·L^−1^·h^−1^, respectively, while enzymatic degradations occurred at a much higher rate of 37 and 66 μmol·L^−1^·h^−1^, respectively. While in the present study, it was visually confirmed that the fruit contained no *Botrytis cinerea*, and thus presumably no laccase. It is possible that other PPOs endogenous to grapes were not inhibited by the 35 mg/L SO_2_ addition at crushing [59]. Therefore, these PPOs may have contributed more significantly than chemical oxidation to the 21% decrease in total phenolics in AirMix wines, when compared to PD wines at pressing. Notably, phenolic compounds themselves may inhibit enzymatic activity, reducing the effect of PPO-mediated oxidation as extraction progressed during maceration [60].

Additionally, the greater binding capacity of oxidized tannins to grape solids in the presence of cell wall material, as well as the reduced water solubility of oxidized tannins, and subsequently greater precipitation out of solution may have contributed to reduced phenolic content in AirMix wines [61,62,63,64].

Previous research using the same AirMix protocol but with bubbles expelled from 2 µm sinter elements reported 49% losses in total phenolics when compared to PD wines. This is somewhat in contrast to the 21% losses and 3.2 mm tubing when also compared to PD wines [8]. Assuming no coalescence and that bubbles exit at the pore diameter, a 2 µm sinter would generate bubbles with a surface area of approximately 0.000013 mm^2^, while 3.2 mm tubing would yield bubbles with a surface area of 32.2 mm^2^. Although air bubble coalescence is inevitable [65], this theoretical outlook emphasizes the substantial difference in gas to liquid contact and consequently oxygen dissolution rates between studies. Therefore, the finer pore size of the previous study [8] may be expected to dissolve oxygen at a faster rate than likely occurred in the AirMix wines of the present study.

However, dissolved oxygen concentration does not directly dictate oxidation-reduction potential (ORP) or the activation of oxygen into reactive species. Previous research determined a 1:1 molar ratio between oxygen consumption and Fe^2+^ consumption, yielding hydrogen peroxide (H_2_O_2_) and Fe^3+^ [33]. H_2_O_2_ may then react with further Fe^2+^, yielding the hydroxyl radical or ferryl ion, FeO^2+^, powerful oxidizers capable of oxidizing phenolic compounds such as tannins and anthocyanins. Therefore, Fe^2+^ regeneration was identified as the rate-determining step in the wine oxidation pathway [66]. While Fe^2+^ and Fe^3+^ were not separately quantified in the present study, Fe cation concentrations at pressing ranged from 1.1 (AirMix) to 2.2 (N_2_Mix, RedoxConN_2_) (Figure S3). Despite the potential for dissolution of up to 7 mg O_2_/L in air saturated must being reported previously [67], only a fraction of the DO concentration can be activated by Fe^2+^ due to its comparatively low concentrations. Following these principles of wine oxidation, it is likely that even the larger bubble size and limited surface area expelled from 3.2 mm tubing allowed for the dissolution of O_2_ sufficient to maximally interact with Fe^2+^, initiating the oxidation cascade. Thus, it follows that by chemical oxidation mechanisms alone, it could be expected that no difference in oxidation of polyphenols may be found despite differences in air bubble size in cases of sufficient mixing and relatively small volume. However, this purely chemical oxidation mechanism does not take into account the potential presence of PPO, which, if present, will further degrade phenolics [68]. This degradation was likely exacerbated in AirMix wines by the excess oxygen, particularly when injected through small-pore gas outlets. This comparison suggests that the physical design of the gas injection system, specifically, pore size, may significantly influence the extent of phenolic loss during fermentation, likely mediated by the presence of PPO, although a direct comparison within a single experiment may be needed to confirm this relationship.

**Figure 7:**
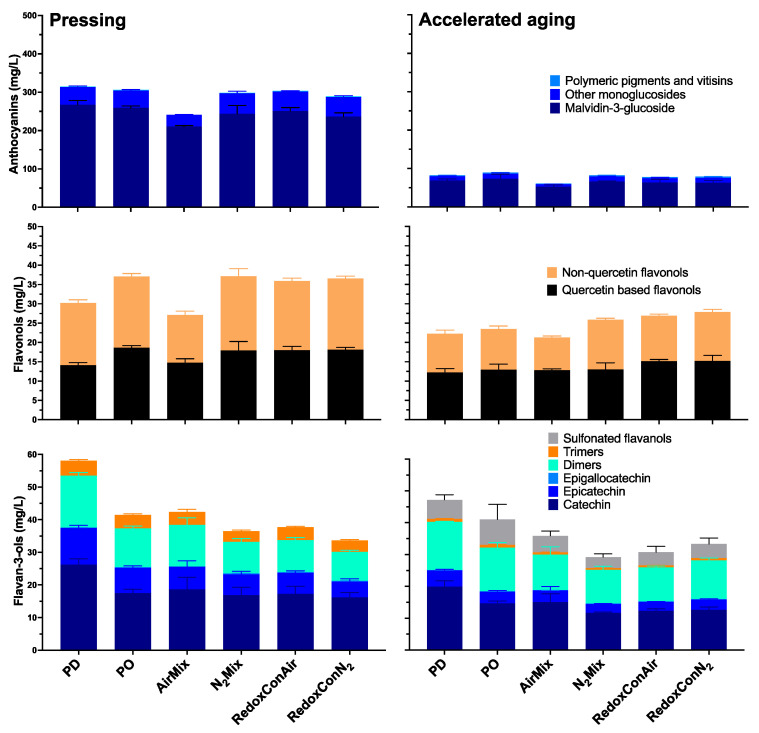
Detailed anthocyanin (**top row**), flavonol (**middle row**), and flavan-3-ol (**bottom row**) composition of Pinot noir wines produced with selected cap management protocols, measured after pressing (**left column**) and accelerated aging (**right column**).

Beyond the broad classes of anthocyanins, tannins, and total phenolics, a more detailed analysis of anthocyanin composition, including several key anthocyanin families, as well as flavonol and flavan-3-ol analyses of the wines are displayed in Figure 7.

Among flavonols, relatively minor differences in both quercetin derived and non-quercetin flavonols were shown among PO, N_2_Mix, RedoxConAir, and RedoxConN_2_ wines. Interestingly, PD wines contained the lowest concentrations of quercetin based flavonols at pressing, suggesting these compounds do not follow the same oxidation trends as is seen in the broader analysis of total phenolics. More significant differences between treatments were shown in non-quercetin flavonols, where, at pressing, 24% lower concentrations were shown in AirMix wines when compared to the next lowest—PD wines (Figure 7). Similar to anthocyanins and tannins, the differences in flavonol concentration between the treatments lessened upon accelerated aging.

Alternatively, the flavan-3-ol compositions of the wines revealed unique outcomes relating physical cap disruptions during alcoholic fermentation to the concentrations of these compounds in the wines, with continued effects lasting into aging and accelerated aging (Figure 7). Punch-downs resulted in 48% more monomeric flavan-3-ols at pressing than pump-overs, including a 50% increase in catechin. Supporting this result, an analysis of cap composition at the end of fermentation (Table S5) showed that PD wines contained 50% fewer intact berries and 17% fewer seeds remaining in the cap by weight, compared to PO wines. This indicates more extensive berry breakage and submergence of seeds into the must in PD wines, likely contributing to the increased flavan-3-ol extraction. While flavan-3-ols are also present in grape skins, they are predominantly concentrated in the seeds [69]. Furthermore, seed phenolics are thought to extract independently of local concentration gradients, while skin phenolics extract at rates dependent on said gradients [16]. Thus, the greater flavan-3-ol concentrations in PD wines can be attributed to enhanced seed submerging due to physical berry breakage, whereas differences in skin phenolics were comparatively minor due to diffusion limitations.

Accelerated aging resulted in a shift in composition of flavan-3-ols among the wines. While no significant concentrations of sulfonated flavan-3-ol adducts were detected in any treatment at pressing, the flavan-3-ol monomers, dimers, and trimers were comprised of between 13% (N_2_Mix) and 24% (PO) sulfonated adducts after accelerated aging, as has been reported due to wine aging previously [70].

The present findings demonstrate that cap management protocols can be employed to target desired phenolic profiles in red wines, with effects observed across anthocyanins, tannins, flavonols, and flavan-3-ols, as well as total phenolics. While aging diminished some of these differences, PD wines maintained increased flavan-3-ol content even after accelerated aging, suggesting increased extraction from the grape seeds in these wines. In contrast, N_2_Mix, RedoxConAir, and RedoxConN_2_ wines achieved comparable phenolic extraction to the traditional cap management techniques of punch-downs and pump-overs in most phenolic classes. The most pronounced phenolic outcomes were observed in AirMix wines, with PPO activity potentially influencing phenolic losses. The AirMix protocol may nonetheless be a valuable tool in the production of early-to-market wine styles, with softened tannins and reduced need for bottle aging. Together, these results suggest that cap management techniques based around gas mixings may allow for varying stylistic choices to be made, while specifically flavan-3-ol extraction may require more physical breakage of the cap and berries.

### 2.6. Volatile Composition of the Wines

The wine’s volatile composition plays a critical role in defining varietal character and wine quality. Previous research has suggested cap management protocols that minimize degassing of CO_2_, especially through limited mixing, may allow for a greater retention of volatile compounds [8]. The same study, under conditions of limited mixing, reported no perceived reduction aroma (empirically attributable to H_2_S and related sulfides), in sensory analysis in wines of the same N_2_Mix protocol three months after bottling. This was attributed to the CO_2_ stripping of highly volatile H_2_S during nitrogen injections, which may have a similar effect on ester concentrations. Additionally, previous research detailed the interaction of phenolic composition of model wines and the related sensory perception of volatile esters, finding that some esters, namely ethyl butyrate and ethyl octanoate doubled or tripled in odor threshold in the presence of catechin, and ethyl octanoate also decreased in volatility [71]. Therefore, cap management effects on extraction and varying extents of CO_2_ degassing may play a role in the aromatic profile of wine—an association that this section aims to elaborate upon.

**Figure 8:**
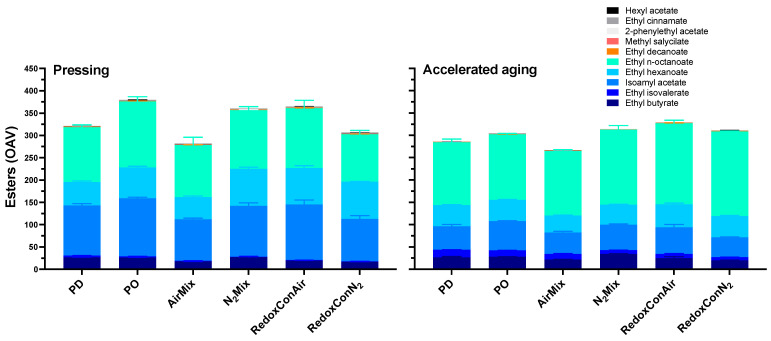
Detailed ester composition at pressing of Pinot noir wines produced with selected cap management protocols, measured after pressing (**left**) and accelerated aging (**right**). Values represent the odor activity value (OAV) of selected esters.

In the present study, the esters ethyl-n-octanoate, isoamyl acetate, and ethyl hexanoate were the primary contributors to the aromatic profiles of all wines, based on their concentrations and odor activity values (OAVs) (Figure 8, Table S3). Of these esters, ethyl n-octanoate showed only minor differences between treatments; however, the typically banana-scented isoamyl acetate ranged in OAV at pressing from 130 and 125 in PO and RedoxConAir wines, to as low as 93 and 94 in AirMix and RedoxConN_2_ wines, respectively.

The third most odor active ester measured in all wines was the pineapple-scented ethyl hexanoate. N_2_Mix, RedoxConAir, and RedoxConN_2_ wines contained the highest concentrations of this compound, each showing roughly 58% more ethyl hexanoate than PD wines at pressing, with OAVs ranging 81 to 83 for in wines of these treatments. Alternatively, PD and AirMix wines showed the lowest concentrations, with OAVs of 50 and 52, respectively.

Among all treatments, AirMix wines contained the lowest concentration of most esters measured and the lowest combined OAV. However, previous research has suggested Pinot noir and Petite Sirah wines produced with this highly oxidative cap management regime produced wines with increased red fruit aroma as perceived in sensory analysis, despite similarly reduced concentration of esters [8]. Informal sensory analysis of the present wines confirmed a similar result. This may be attributed to the increase in the odor threshold of many esters including ethyl octanoate and ethyl butyrate in the presence of catechin, as described in previous research [71]. Considering these are the first and fourth most odor active compounds in the wines at pressing, it is hypothesized that the reduced phenolic content of AirMix wines may have contributed to an increased perception of fruitiness. This outcome underscores the complexity of the chemical and volatile matrices that shape wine aroma.

Upon accelerated aging, isoamyl acetate concentration decreased more than all other esters measured. At pressing this compound represented between 56% (AirMix) to 60% (PD) of the total measured volatile concentration of the wines. After accelerated aging, however, it comprised only 36% in RedoxConN_2_ wines to 47% in PO wines.

Interestingly, while the total concentration of measured volatiles reduced significantly, ethyl n-octanoate and ethyl isovalerate increased. Ethyl isovalerate was a minor contributor to the aroma profile of the wines at pressing, with OAVs below 4 in all wines. However, after accelerated aging, this compound ranged from 7 in RedoxConN_2_ wines to 17 in PD wines.

**Figure 9:**
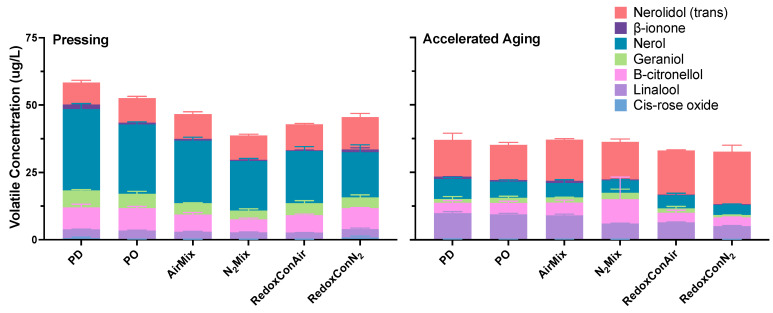
Terpenoid and norisoprenoid concentrations at pressing of Pinot noir wines produced with selected cap management protocols, measured after pressing (**left**) and accelerated aging (**right**).

Unlike esters, all terpenes and norisoprenoids measured were below detection threshold, except for β-ionone (Figure 9; Table S3). For this reason, Figure 9 details the concentrations of selected terpenoids, rather than OAV. β-Ionone, a norisoprenoid, ranged in OAVs from 3 to 19 in N_2_Mix and PD wines at pressing, respectively. While these OAVs show the significant contribution to the wines aromatic profile, β-ionone was near the lowest in concentration of all volatiles measured. However, the very low odor threshold of 0.09 µg/L [72] allowed for even small variations in concentration to impart significant differences in OAV between the treatments.

After accelerated aging, trans-nerolidol increased in all treatments, with the largest increase being in RedoxConAir wines which showed 69% greater concentrations than at pressing. Simultaneous decreases occurred in nerol and geraniol concentrations in all wines. Previous research has shown the decrease of nerol and geraniol during accelerated aging of Valpolicella wines and the paired formation of terpineol, among other cyclic terpenoids that were not measured in the present study [73] but may have contributed to the aromatic development of these wines during aging.

Together, the volatile chemistry of the wines demonstrated that cap management regimes may produce a measurable influence on wine aroma, with these effects being shown quite distinctly in some volatile compounds, namely, isoamyl acetate. Notably, both the highly oxidative AirMix wines and the reductive, minimally degassed RedoxConN_2_ wines contained significantly lower concentrations of isoamyl acetate than all other treatments. While low concentrations of this ester in AirMix wines may be explained by oxidative degradation and CO_2_ degassing and stripping, RedoxConN_2_ wines likely suffered a different mechanism. The weak and still active fermentation at pressing in RedoxConN_2_ wines may have simply not biosynthesized esters to the same extent as other treatments by the time of pressing [74], effectively offsetting any increased retention from reduced CO_2_ stripping.

Lastly, it should be noted that in previous studies, 20 mg/L glutathione additions at bottling resulted in increased preservation of volatile compounds [42]. However, in the present study, no correlation was found relating GSH concentration at pressing with changes in the measured volatile concentration between pressing and accelerated aging.

## 3. Materials and Methods

### 3.1. Grapes

Pinot noir clone 828 (1.58 t) was harvested by hand on 9 September 2024, from Drum Canyon Vineyard in the Sta. Rita Hills AVA, California (Lompoc, CA, USA). Fruit was transported to the Research Winery of the Wine and Viticulture Department (California Polytechnic State University, San Luis Obispo, CA, USA) and processed on the same day as fruit harvesting. Visual inspection of the fruit confirmed negligible presence of *Botryitis cinerea*.

Basic fruit chemistry and berry compositional data are shown in Table S4. Fruit chemistry was assessed using a composite sample made up of 100 mL from each fermentation vessel immediately after crushing, destemming, and sufficient homogenization. Analyses included titratable acidity (TA), malic acid, yeast assimilable nitrogen (YAN), copper ions, and iron ions, which were measured enzymatically or spectrophotometrically (SPICA Automatic Analyzer, Admeo, Angwin, CA, USA) using commercially available kits (Biosystems, Barcelona, Spain). Total soluble solids (Brix) were measured using a temperature-compensated densimeter (DMA, Anton Paar, Graz, Austria), and pH was measured using a pH probe (Thermo Fisher Scientific, Waltham, MA, USA).

Berry compositional data and the liquid:solid ratio was collected following previously described methods [10]. Post-fermentative berry composition (Table S5) was assessed by collecting 3.8 L of grape solids from various cap depths prior to pressing. From this, 50 g subsamples were used to separate and weigh whole berries and seeds present in the cap.

### 3.2. Winemaking and Experimental Design

Upon receiving the grapes in 0.5 t Macro-Bin containers (IPL Macro, Fairfield, CA, USA), the fruit was weighed using an industrial scale (Cardinal Detecto, Model 204, Webb City, MO, USA) and subsequently destemmed and crushed using a destemmer–crusher unit (Bucher Vaslin, Zurich, Switzerland). The must was then evenly distributed by weight into eighteen 110 L stainless steel cylindrical tanks, each with interior dimensions of 89 cm in height and 40 cm in diameter (Westec Tank & Equipment, Healdsburg, CA, USA). The 18 tanks were randomly sorted into six groups of three, forming the six cap management treatments, each conducted in triplicate (*n* = 3). Sulfur dioxide (SO_2_, 35 mg/L) was added post-crushing, followed by homogenization of the must with a punch-down tool.

All wines were inoculated with 20 g/hL *Saccharomyces cerevisiae* selected dry yeast (strain RC 212, Lallemand, Montreal, QC, Canada) 3 h after SO_2_ addition. Briefly, 30 g/hL of rehydration nutrient was added to the yeast and water mixture (Go-Ferm Sterol Flash, Lallemand, Rexdale, ON, Canada), and mid-fermentation nutrient additions were subsequently carried out as needed in all treatments (Fermaid K, Lallemand, Rexdale, ON, Canada). Maceration time was set to 10 days, and temperatures were maintained at an average of 23 °C during this period. Temperature was controlled by circulating hot or cool propylene glycol through the jacket of the fermentation vessels as needed and was measured on each tank continuously throughout the maceration period with a wireless temperature probe (Elitech RC-5, Bruker, Billerica, MA, USA) (Supplemental Figure S1). Similarly, ORP was measured during fermentation of all treatments using a bright platinum electrode and silver/silver chloride (Ag/AgCl) reference electrode (EasyFerm Plus ORP Arc 120, Hamilton, Reno, NV, USA) from which ~220 mV is subtracted from values referenced to a standard hydrogen electrode (SHE). Following the probe manufacturer’s guidelines, all probes were calibrated prior to use and were cleaned and stored accordingly after use, resulting in no probe fouling in the present study, although fouling may be of concern in the use of ORP probes [75]. The probe was placed in the liquid must, 38 cm above the bottom of the tank, and below the cap. ORP probes recorded the ORP of the must throughout the entirety of maceration at one-minute intervals.

PD, PO, AirMix, and N_2_Mix treatments were composed of two cap management interventions per day in the morning and evening, while RedoxConAir and RedoxConN_2_ treatments followed continuously automated protocols throughout maceration. Punch-down (PD) wines were gently mixed with a stainless-steel punch down tool for three minutes. Pump-overs (PO) were performed by draining juice or wine out of the side valve of the tank into a plastic bin open to the air. Simultaneously, a pneumatic diaphragm pump (Wilden, Grand Terrace, CA, USA) transferred the wine through 3.81 cm hosing to the top of the tank, effectively wetting the cap. Each PO intervention was conducted for three minutes, amounting to two full volumes of wine being pumped over at each session. AirMix and N_2_Mix wines were produced by injecting compressed air or N_2_, respectively, into the bottom of the fermentation tanks through a 3.2 mm inside-diameter plastic tubing (Uline, Pleasant Prairie, WI, USA). The gas outlet was firmly held at 5 cm above the bottom of the tank. RedoxConAir wines were maintained above a minimum ORP of −40 mV using a custom programmable logic controller (PLC) (MeshVines, Davis, CA, USA) programmed to inject air for 10 s whenever ORP dropped below the −40 mV setpoint. Each RedoxConN_2_ wine was paired with a corresponding RedoxConAir replicate and received nitrogen gas injections simultaneously. Because N_2_ does not affect ORP, these pairings intended to account for the physical effects of gas mixing without introducing oxygen or altering redox potential. RedoxConAir and RedoxConN_2_ wines received a 1 h injection of air and nitrogen, respectively, on day two to encourage the onset of alcoholic fermentation. For all air or N_2_ injection treatments, gas flow rate was maintained at 3.5 L of gas per min. Gas flow was regulated using a flow meter (Model 351, Harris Products Group, Mason, OH, USA) and sourced from an air compressor (Ingersoll Rand, Davidson, NC, USA) or food grade N_2_ gas cylinder (Matheson, Irving, TX, USA). Visual examination confirmed that the small bubble size and relatively low flow rate did not disrupt the integrity of the cap during gas mixing sessions.

#### Alcoholic Fermentation and Bottling

**Figure 10:**
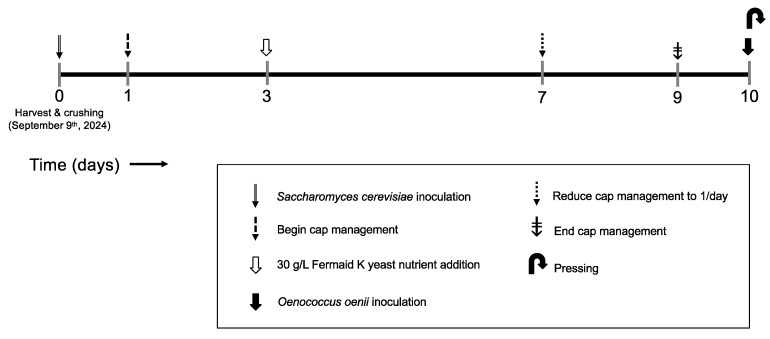
Timeline of experimental design during alcoholic fermentation and maceration of Pinot noir wines.

An overview of the experimental design applied during alcoholic fermentation is shown in Figure 10. Immediately after crushing, all wines received a 2.5 L acidified (4 g/L tartaric acid) water addition. These additions to each tank ameliorated high fermentable sugar content and thus potential alcohol. At pressing, all wines were inoculated with *Oenococcus oenii* malolactic bacteria (MLB) (Lalvin VP41, Lallemand, Rexdale, ON, Canada). Delaying malolactic fermentation until the end of alcoholic fermentation ensured that the oxidation-reduction potential (ORP) data reflected only the activity of *Saccharomyces cerevisiae*, without the influence of concurrent malolactic fermentation. Extraction during maceration was managed according to commercial standard practices, with cap management reduced to one session per day after day 7 of maceration and subsequently to zero cap management sessions per day on day 9 (Figure 10). Maceration time was set to 10 days to follow industry standard practices for red wines, achieving phenolic and aromatic extraction from grape solids without the effects of extended maceration described previously [11,12,43].

Upon completion of malolactic fermentation, all wines were adjusted to 0.3 mg/L molecular SO_2_ and stored at 8 °C until filtering through a plate and frame filter (Super Jet, Buon Vino, Cambridge, ON, Canada) with 2 µm cellulose filter pads (Vintners Vault, Paso Robles, CA, USA). Immediately after filtering, all treatments were readjusted to 0.3 mg/L molecular SO_2_ and bottled in 750 mL glass bottles under argon and corked using technical corks (Diam 30, 49 mm height, 23.5 mm diameter, G3 Enterprises, Modesto, CA, USA). Bottled wines were stored in a vertical position and kept in cellar-like conditions (12 to 14 °C) until selected moments of analysis during aging.

Accelerated aging was performed following a previously published protocol [10], whereby wines were bottled in 50 mL air-tight glass ampules (DWK Life Sciences, Melville, NJ, USA) and incubated for a 5-week period at 38 °C (Heratherm 120 V incubator, Thermo Fisher Scientific, Waltham, MA, USA).

### 3.3. Wine Analyses

Prior to analysis, all wine samples were centrifuged for 8 min at 15,000× *g* using a microcentrifuge (Eppendorf Model 5415D, Hamburg, Germany). For samples still actively fermenting, centrifugation was carried out at 2 °C. The resulting supernatant was then carefully transferred into clean 1-mL Eppendorf tubes.

#### 3.3.1. Chemical Composition

Wine chemistry was measured following the same methods as described for juice chemistry, with the additional analyses of acetic acid, lactic acid, free SO_2_, total SO_2_, and acetaldehyde, which were measured enzymatically as well as iron and copper ions measured by colorimetry (Spica, Admeo, Angwin, CA, USA) using commercially available kits (Biosystems, Barcelona, Spain). Ethanol content (% *v*/*v*) was measured with near-infrared spectroscopy using an Alcolyzer Wine M analysis system (Anton Paar, Graz, Austria).

#### 3.3.2. Wine Spectrophotometric Analysis

Anthocyanins, tannins, polymeric pigments, total phenolics, and full visible spectrum absorbances were monitored throughout the winemaking process and into bottle aging and accelerated aging. Spectrophotometric methods were used to assess phenolic composition and color metrics and to collect full visible spectrum absorbance profiles. All measurements were conducted using a Cary 60 UV–Vis spectrophotometer equipped with an 18-sample cell auto-sampler (Agilent Technologies, Santa Clara, CA, USA). Anthocyanins are expressed as mg/L malvidin-3-glucoside, total phenolics are expressed as mg/L gallic acid equivalents, and total polymeric pigments were measured as previously described [76]. Tannin content was determined via protein precipitation and reported as (+)-catechin equivalents (CE). Absorbance scans and color measurements were carried out in 1 mm path-length quartz cuvettes using Cary WinUV Color software (Version 6.0, Startek Technology, Denver, CO, USA).

#### 3.3.3. Anthocyanins, Anthocyanin-Derived Pigments, Flavonols, and the Grape Reaction Product

Wines were analyzed by HPLC-diode array detector (DAD) at selected time-points of alcoholic fermentation, bottle aging, and accelerated aging using an Agilent 1100 series HPLC system coupled to a DAD (Agilent Technologies, Santa Clara, CA, USA), as previously described [77], with minor modifications. The HPLC separation was conducted using a solvent gradient consisting of 5% formic acid in water (Solvent A) and pure methanol (Solvent B), with a flow rate of 1 mL min^−1^. The gradient profile was programmed as follows: 0 min at 23% B, 5 min at 26% B, 15 min at 60% B, and 16 min at 100% B. Each run used a 25 μL injection volume. Separation occurred on an Agilent Zorbax Eclipse Plus C18 column (4.6 × 100 mm, 3.5 μm; Agilent Technologies, Santa Clara, CA, USA), maintained at 40 °C and fitted with a guard column packed with the same stationary phase. The column was equilibrated for 2 min at 23% Solvent B before sample injection. Photodiode array detection (DAD) was performed from 210 to 600 nm, and the quantification and identification of anthocyanins as well as flavonols and 2-S-glutathionyl caftaric acid were carried out by peak area measurements and absorbance spectra at 520 and 356 nm, respectively. All solvents used were HPLC grade and sourced from Fisher Scientific (Hampton, NH, USA). Monomeric anthocyanins were quantified against a malvidin-3-glucoside chloride standard (Extrasynthèse, Lyon, France) using a calibration curve with R^2^ = 0.99. To facilitate interpretation, pigments were quantified in malvidin-3-glucoside equivalents and grouped as malvidin-3-glucoside and malvidin derivatives; other glycosides (i.e., monoglucosylated anthocyanins, including delphinidin-, cyanidin-, petunidin-, and peonidin-3-glucosides); and polymeric pigments and vitisins (i.e., pyranoanthocyanins, including vitisin A and vitisin B, as well as polymeric pigments). Flavonols were quantified using quercetin-3-glucoside (Sigma-Aldrich, St. Louis, MO, USA), as standard and a standard calibration curve (R^2^ = 0.99). Flavonols were quantified in quercetin-3-glucoside equivalents and grouped as quercetin derivatives (i.e., quercetin-3-glucoside, quercetin-3-gluconoride, and quercetin aglycone) or other flavonols (i.e., myricetin, laricitrin, isorhamnetin, syringetin, kaempferol, and their respective derivatives). Retention times for these compounds are found in Table S6.

#### 3.3.4. Monomeric Flavan-3-ols

Wine samples were analyzed at selected time points of aging on an Agilent 1260 Infinity II liquid chromatograph (HPLC) coupled to an Agilent Ultivo MS scan from 100 to 1400 *m*/*z* (Agilent Technologies, Santa Clara, CA, USA) following previously described methods [70]. A 10 μL aliquot of each sample was injected onto an Agilent Zorbax Eclipse Plus C18 column (4.6 × 100 mm, 3.5 µm; Agilent Technologies). The mobile phases were (a) 0.1% formic acid in water and (B) 0.1% formic acid in acetonitrile. The solvent flow rate was maintained at 0.8 mL min^−1^, and the column temperature was held at 25 °C. The gradient was as follows: 0 min, 3% B; 2 min, 3% B; 10 min, 6% B; 25 min, 42% B; 30 min, 100% B; 32.5 min, 3% B. Samples in the autosampler tray were held at 10 °C. Samples were run in negative mode using an Agilent dual electrospray ionization (ESI) source (Agilent Technologies, Santa Clara, CA, USA). Source conditions were as follows: drying gas at 11.0 L/min and 325 °C, nebulizer pressure at 35 psi, capillary voltage at 3000 V, and fragmentor voltage set to 130 V. Full-scan MS data were acquired across a mass range of *m*/*z* 100 to 2000. Instrument calibration was performed prior to each analytical sequence in accordance with the manufacturer’s recommended protocol. Flavan-3-ols are measured in catechin equivalents, and measured compounds included (+)-catechin, (−)-epicatechin, (−)-epicatechin gallate, (−)-epigallocatechin, dimers (i.e., catechin-catechin 1, catechin-catechin 2, catechin-catechin 3, catechin-catechin 4, catechin-catechin 5, A type 1, A type 2, A type 3, A type 4, and catechin-catechin gallate), trimers (i.e., catechin-catechin-catechin 1, catechin-catechin-catechin 2, and catechin-catechin-catechin 3), and sulfonated flavan-3-ols (i.e catechin-SO_3_H 1, catechin-SO_3_H 2, catechin-SO_3_H 3, catechin-SO_3_H 4, catechin-SO_3_H 5, catechin-SO_3_H 6, gallocatechin-SO_3_H, catechin-catechin-SO_3_H 1, catechin-catechin-SO_3_H 2, gallocatechin-gallocatechin-SO_3_H 1, gallocatechin-gallocatechin-SO_3_H 2, gallocatechin-catechin-SO_3_H 1, gallocatechin-catechin-SO_3_H 2, gallocatechin-catechin-SO_3_H 3, and gallocatechin-catechin-SO_3_H 4). Retention times for these compounds are found in Table S7. All flavan-3-ols were quantified in catechin equivalents.

#### 3.3.5. Glutathione

Glutathione and glutathione disulfide were measured following previously described methods [78]. Juice and wine samples were analyzed at selected time points of aging on an Agilent 1260 Infinity II liquid chromatograph (HPLC) coupled to an Agilent Ultivo MS (Agilent Technologies, Santa Clara, CA, USA). A 2 μL aliquot of each sample was injected onto an Agilent Zorbax Eclipse Plus C18 column (4.6 × 100 mm, 3.5 µm; Agilent Technologies). The mobile phases were (A) water with 0.1% formic acid and (B) acetonitrile with 0.1% formic acid at a flow rate of 1 mL min^−1^. The solvent flow rate was maintained at 0.6 mL min^−1^, and the column temperature was held at 40 °C. The elution gradient was as follows: 2% of B from 0 to 0.6 min, 2–10% of B from 0.6 to 5 min, 10–100% of B from 5 to 6 min, and 100% of B from 6 to 8 min. The column was equilibrated for 3 min with 2% of B. Detection was performed by multiple reaction monitoring. The electrospray positive ionization mode (ESI+) was applied using the following source parameters: gas temperature at 300 °C, gas flow at 5 L min^−1^, nebulizer at 30 psi, and capillary voltage at 4500 V. The fragmentor voltage and collision energy were optimized with the standards separately and were 119 V and 19 V, respectively, for GSH and 110 V and 7 V, respectively, for GSSG. Quantification was performed using the following transitions: 308 *m*/*z* → 179 *m*/*z* for GSH and 613 *m*/*z* → 355 *m*/*z* for GSSG. The linearity of the LC-MS/MS method was calculated in the range of 1–20 mg L^−1^ for GSH and GSSG. Instrument calibration was performed prior to each analytical sequence in accordance with the manufacturer’s recommended protocol.

#### 3.3.6. Volatile Compounds

Volatile compounds were extracted from wine samples using stir bar sorptive extraction (SBSE) with polydimethylsiloxane (PDMS)-coated stir bars (Twister^®^, Gerstel, Germany), following a previously described methodology [79,80]. The extraction was performed by direct immersion of the Twister^®^ stir bars into the wine, with stirring at 1000 rpm for 1 h at ambient temperature. Following extraction, volatiles were thermally desorbed using an automated thermal desorption unit (TDU2, Gerstel, Germany) coupled to a cooled injection system (CIS-4, Gerstel, Germany), equipped with a liner packed with 20 mg of Tenax TA^®^. Thermal desorption conditions and chromatographic conditions followed previously described methods [79]. The desorbed volatile compounds were separated in an Agilent 8890 gas chromatograph system (GC) coupled to a triple quadrupole (QqQ) Agilent 7000D mass spectrometer (Agilent Technologies), operating in simple quadrupole (Q) mode. Quantification was performed according to calibration curves obtained using pure standards diluted into synthetic wine (13.5% ethanol, 5 g/L tartaric acid, pH 3.6, 20 mg SO_2_/L). Calibration of internal standards for the following compounds were prepared using pure standards (>95%) (Table S8): ethyl butyrate, ethyl isovalerate, isoamyl acetate, ethyl hexanoate, hexyl acetate, ethyl n-octanoate, ethyl decanoate, methyl salicylate, 2-phenylethyl acetate, ethyl cinnamate, geraniol, cis-rose oxide, linalool, β-citronellol, nerol, β-ionone, and nerolidol (trans). Compounds not included in the calibration were identified using mass spectra from the NIST library and quantified as equivalents from the internal standard γ-hexalactone. Odor activity values were calculated based on odor thresholds determined in previous work [72,81,82,83,84,85,86].

#### 3.3.7. Data Analysis

Plots of sugar consumption (Brix), temperature, ORP evolution, and time-course extraction of phenolic compounds during winemaking and aging were produced using Graphpad Prism software 10.3.0 (Graphpad, La Jolla, CA, USA). Moving averages of ORP were calculated with a 0th order smoothing polynomial and averaging of 500 neighboring data points on each side, using Graphpad Prism software 10.3.0. A one-way analysis of variance (ANOVA) was conducted (*p* < 0.05) on the basic chemical composition, phenolic composition, and volatile chemistry of the wines. XLSTAT 2023.3.1 (Addinsoft, Version 2023.1414, Paris, France) was used for all statistical analyses.

## 4. Conclusions

The present study investigated six contrasting cap management protocols in the production of Pinot noir wines, including the widely employed punch-downs and pump-overs, as well as four modalities of fully automated gas mixing protocols, namely, air or N_2_ gas injections for 1 h, twice daily (AirMix; N_2_Mix), 10-second air injections if the ORP dropped below −40 mV (RedoxConAir), and a treatment comprised of 10-second N_2_ injections if the respective replicate of RedoxConAir wines received an air injection. The gas injection protocols were entirely automated using a programmable logic controller equipped with gas solenoids, and these wines required no manual cap management. The oxidation-reduction potential (ORP) was monitored continuously during alcoholic fermentation, highlighting how cap management may be designed to produce predictable ORP evolutions in wine. This study aimed to elucidate the effects of cap management and ORP evolution on the phenolic, volatile, and chemical composition, as well as the aging dynamics of red wine, with the following major takeaways.

The treatments that maintained lower ORPs during alcoholic fermentation (N_2_Mix, RedoxConN_2_) preserved more GSH, whereas the oxidatively produced AirMix wines depleted nearly all GSH, in turn effecting the GSH:GSSG ratio with potential implications on wine aroma and color during aging [40,41,87]. In contrast, no relationship was observed between ORP evolution during alcoholic fermentation and the phenolic composition of the wines. This may be because ORP is likely regulated by a limited number of influential redox couples that do not directly affect phenolic extraction or oxidation. We therefore suggest that the ORP’s use in wine fermentation may be more suited to managing yeast health and performance, modeling predictive fermentation kinetics, and minimizing volatile sulfur compound production [23,24,25,88]. Despite a lack of direct correlation between ORP and phenolic oxidation, the high volume of air injected in the AirMix protocol resulted in phenolic losses. Such a method may present a viable alternative to post-fermentative micro-oxygenation (MOX) treatments [89,90]. MOX treatments have been shown to result in increased acetaldehyde, likely due to the proliferation of unwanted spoilage yeast and bacteria in the presence of oxygen [91,92]. In contrast, implementing oxygenations during alcoholic fermentation, such as the AirMix protocol of the present study, may alternatively allow *Saccharomyces cerevisiae* to provide a competitive factor and promote an anaerobic environment.

Under the conditions of this study, that is, of fermentation vessels of relatively small volume and favorable geometry, gas mixing techniques yielded promising results as alternatives to traditional cap management. However, commercial-scale fermentations may require additional measures, such as targeted glycol cooling to manage cap temperature effectively. While further scaling studies are warranted, the present findings suggest that using gas injections, cap management may be automated or potentially incorporated in conjunction with punch-downs or pump-overs to meet stylistic goals. These results display the potential for technology-driven approaches to deliver stylistic diversity which may be useful in both blending and wine portfolio diversification.

## Data Availability

The original contributions presented in this study are included in the article. Further inquiries can be directed to the corresponding author(s).

## Supplementary Materials

The following supporting information can be downloaded at: https://www.mdpi.com/article/10.3390/molecules30153172/s1, Table S1. One-way analysis of variance (ANOVA) of the basic chemical composition of Pinot noir wines produced with selected cap management protocols. Values represent the mean of three tank replicates (*n* = 3); Table S2. One-way analyses of variance (ANOVA) of the anthocyanin, tannin, polymeric pigment, and total phenolic composition of Pinot noir wines produced with selected cap management treatments at selected time points during winemaking and bottle aging. Values represent the mean of three tank replicates (*n* = 3); Table S3. One way analysis of variance (ANOVA) of odor activity values (OAV) of the volatile composition of Pinot noir wines produced with selected cap management protocols. Values represent the mean OAV of three tank replicates (*n* = 3), measured at selected time points during winemaking and aging; Table S4. Basic juice chemistry of Pinot noir grapes on the day of harvesting; Table S5. One-way analyses of variance (ANOVA) of the composition of the cap after alcoholic fermentation of Pinot noir wines produced with selected cap management protocols. Values represent the mean of three tank replicates (*n* = 3); Table S6. Retention times of selected anthocyanins and flavonols identified in Pinot noir wines by HPLC-DAD; Table S7. Retention times of selected monomeric flavan-3-ols, dimers, and trimers identified in Pinot noir wines by LCMS; Table S8. Retention times, regression equations, and r^2^ values obtained for selected volatile aroma compounds as measured with GC/MS using pure standards (>95%); Figure S1. Temperature during alcoholic fermentation of Pinot noir wines made with varying cap management protocols. Each data point represents the mean of three replicates (*n* = 3) with shading represents the standard deviation of these replicates; Figure S2. Full visible absorption spectrum scans of Pinot noir wines produced with selected cap management protocols, measured at selected time points of winemaking and aging. Data represents the mean of three replicates (*n* = 3). Figure S3. Iron (Fe) and copper (Cu) cation concentrations of Pinot noir wines produced with selected cap management protocols, measured at pressing. Data represents the mean of the replicates (*n* = 3), and error bars represent the standard error of the mean.

## Abbreviations

The following abbreviations are used in this manuscript:

Ag/AgCl	silver/silver chloride
AirMix	air mixing
ANOVA	analysis of variance
CE	catechin equivalents
DO	dissolved oxygen
GRP	grape reaction product (C_23_H_27_N_3_O_15_S)
GRP2	grape reaction product 2 (C_33_H_42_N_6_O_21_S_2_)
GSH	glutathione
GSSG	glutathione disulfide
N_2_Mix	nitrogen mixing
OAV	odor activity value
ORP	oxidation-reduction potential
PD	punch-down
PO	pump-over
PPO	polyphenol oxidase
RedoxConAir	redox control with air
RedoxConN_2_	nitrogen gas mixing paired to RedoxConAir
SHE	standard hydrogen electrode
TA	titratable acidity
YAN	yeast assimilable nitrogen
