# Supplementary material for: Effect of Contrasting Redox Potential Evolutions and Cap Management Techniques on the Chemical Composition of Red Wine
Source: Molecules. 2025 Jul 29;30(15):3172. doi: 10.3390/molecules30153172 (PMC12348074; doi:10.3390/molecules30153172)
Supplement: Supplementary file 1 [file molecules-30-03172-s001.zip › molecules-3717618-supplementary.pdf]

Table S1. One-way analysis of variance (ANOVA) of the basic chemical composition of Pinot noir wines produced with selected cap management protocols. Values represent the mean of three tank replicates (n = 3).

Sampling stage	Maceration treatment	pH	Titratable acidity (g/L tartaric acid)	Ethanol (% v/v)	Glucose + fructose (g/L)	Lactic acid (g/L)	Malic acid (g/L)	Acetic acid (g/L)	Acetaldehyde (mg/L)	Free SO2 (mg/L)	Total SO2 (mg/L)
Pressing	PD	3.49 a	7.56 a	14.78 a	0.36 c	0.11 bc	2.50 a	0.38 a	16.00 b	3.00 a	0.69 b
	PO	3.50 a	7.89 a	14.05 bc	4.92 c	0.12 bc	2.71 a	0.41 a	17.00 b	1.33 a	0.41 b
	AirMix	3.51 a	7.46 a	14.47 ab	3.48 c	0.10 c	2.56 a	0.38 a	19.67 ab	2.67 a	0.33 b
	N_2Mix	3.46 a	7.65 a	13.73 c	20.88 b	0.14 abc	2.53 a	0.32 ab	19.33 ab	1.00 a	1.33 b
	RedoxConAir	3.50 a	7.45 a	13.50 c	21.22 b	0.18 ab	2.75 a	0.28 b	20.00 ab	2.33 a	2.33 b
	RedoxConN_2	3.46 a	7.58 a	12.67 d	43.31 a	0.18 a	2.73 a	0.34 ab	21.67 a	0.67 a	5.00 a
	p	0.3372	0.0954	**<0.0001**	**<0.0001**	**0.0056**	0.2800	**0.0091**	**0.0091**	0.3837	**0.0005**
Bottling	PD	3.55 a	6.71 a	14.63 a	0.48 c	1.12 b	0.02 a	0.46 bc	6.33 a	22.33 a	36.67 a
	PO	3.60 a	6.93 a	14.22 a	0.97 c	1.24 ab	0.02 a	0.53 ab	5.00 a	23.33 a	35.33 a
	AirMix	3.68 a	6.72 a	14.61 a	0.91 c	1.17 ab	0.02 a	0.51 abc	8.00 a	19.00 a	36.00 a
	N_2Mix	3.61 a	6.51 a	14.44 a	3.71 ab	1.19 ab	0.02 a	0.45 c	3.67 a	19.00 a	29.67 a
	RedoxConAir	3.61 a	6.56 a	14.40 a	2.89 b	1.31 a	0.01 a	0.48 abc	8.00 a	20.33 a	37.67 a
	RedoxConN_2	3.57 a	6.73 a	14.35 a	4.34 a	1.27 ab	0.02 a	0.53 a	4.00 a	19.00 a	37.67 a
	p	0.1006	0.6111	0.0762	**<0.0001**	**0.0345**	0.7847	**0.0070**	0.2565	0.6269	0.1296

(*) Different letters within a column for each sampling stage indicate significant differences for Fisher's LSD test and $p < 0.05$. Significant p-values are shown in bold fonts

Table S2. One-way analyses of variance (ANOVA) of the anthocyanin, tannin, polymeric pigment, and total phenolic composition of Pinot noir wines made with selected cap management treatments, at selected time points during winemaking and bottle aging. Values represent the mean of three tank replicates (n = 3).

Sampling Stage	Maceration treatment	Anthocyanins (mg/L mv-3-glu)	Tannins (mg/L)	Polymeric pigments (mg/L)	Total phenolics (mg/L)
Pressing	PD	407 a	627 a	1.5 a	1275 ab
	PO	410 a	609 a	1.1 abc	1281 ab
	AirMix	310 b	552 a	1.3 ab	1006 b
	N_2Mix	425 a	624 a	0.9 c	1348 a
	RedoxConAir	419 a	611 a	1.1 bc	1306 ab
	RedoxConN_2	454 a	642 a	1.0 bc	1390 a
	p	**0.0002**	0.5726	**0.0036**	**0.0198**
Bottling	PD	274 ab	481 a	1.5 a	1088 a
	PO	303 a	486 a	1.2 a	1125 a
	AirMix	229 b	455 a	1.4 a	921 a
	N_2Mix	326 a	492 a	1.3 ab	1129 a
	RedoxConAir	321 a	555 a	1.6 a	1217 a
	RedoxConN_2	320 a	526 a	1.6 a	1247 a
	p	**0.0037**	0.4833	0.1083	0.0921
Accelerated Aging	PD	163 a	433 a	1.7 ab	1199 a
	PO	167 a	416 a	1.6 b	1290 a
	AirMix	121 a	411 a	1.8 ab	1049 a
	N_2Mix	170 a	424 a	1.9 ab	1246 a
	RedoxConAir	164 a	487 a	2.2 a	1293 a
	RedoxConN_2	172 a	475 a	2.2 ab	1339 a
	p	0.062	0.5601	**0.0317**	0.1414

*Different letters within a column for each sampling stage indicate significant differences for Fisher's LSD test and $p < 0.05$. Significant p values are shown in bold fonts.

Table S3. One way analysis of variance (ANOVA) of Odor Activity Values (OAV) of the volatile composition of Pinot noir wines made with selected cap management protocols. Values represent the mean OAV of three tank replicates (n = 3), measured at selected time points during winemaking and aging.

Sampling stage	Maceration treatment	Ethyl butyrate	Ethyl isovalerate	Isoamyl acetate	Ethyl hexanoate	Ethyl n-octanoate	Ethyl decanoate	Methyl salicylate	2-Phenylethyl acetate	Ethyl cinnamate	Hexyl acetate	Cis-rose oxide	Linalool	β-citronellol	Geraniol	Nerol	β-ionone
Pressing	PD	27 a	3.7 a	113 ab	52.3 b	123 a	1.0 a	0.0 a	0.2 b	1.1 a	0.4 b	0.0 a	0.1 a	0.3 ab	0.2 a	0.3 a	19 a
	PO	26 a	2.7 ab	130 a	69.4 a	148 a	1.0 a	0.0 a	0.4 a	1.1 a	0.6 ab	0.0 a	0.1 a	0.3 a	0.2 a	0.3 ab	7 ab
	AirMix	18 b	1.7 bc	93 b	49.6 b	117 a	0.9 a	0.0 a	0.4 a	0.9 a	0.7 ab	0.0 a	0.1 a	0.2 ab	0.1 a	0.3 c	8 ab
	N_2Mix	27 a	1.1 c	114 ab	83.0 a	131 a	1.0 a	0.0 a	0.5 a	1.0 a	0.7 ab	0.0 a	0.1 a	0.2 b	0.1 a	0.2 c	4 ab
	RedoxConAir	20 b	1.2 c	125 a	81.4 a	134 a	1.0 a	0.0 a	0.5 a	1.2 a	0.9 a	0.0 a	0.1 a	0.2 ab	0.1 a	0.2 c	3 b
	RedoxConN_2	17 b	1.0 c	95 b	83.5 a	106 a	0.8 a	0.0 a	0.4 a	1.4 a	0.9 a	0.0 a	0.1 a	0.3 ab	0.1 a	0.2 c	11 ab
	p	**<0.0001**	**<0.0001**	**0.0061**	**<0.0001**	0.2983	0.6411	0.5360	**<0.0001**	0.2140	**0.0135**	0.5404	0.2041	**0.0322**	0.1490	**0.0010**	**0.0473**
3-month bottle aging	PD	25 cd	11 a	63 c	46 b	114 d	0.6 b	0.0 b	0.1 c	0.9 ab	0.1 b	0.0 a	0.4 a	0.2 ab	0.1 a	0.0 ab	9 a
	PO	28 bcd	9 ab	83 abc	47 b	128 cd	0.7 ab	0.0 ab	0.2 ab	1.0 ab	0.2 ab	0.0 a	0.3 a	0.2 ab	0.1 a	0.0 b	3 ab
	AirMix	23 d	8 abc	64 bc	43 b	151 cd	0.6 ab	0.0 ab	0.2 bc	0.8 b	0.2 ab	0.0 a	0.3 a	0.2 ab	0.1 a	0.0 ab	5 ab
	N_2Mix	39 a	6 c	80 abc	50 b	168 bc	0.8 ab	0.0 b	0.3 a	1.0 ab	0.2 ab	0.0 a	0.3 a	0.1 b	0.1 a	0.0 a	3 ab
	RedoxConAir	33 abc	7 bc	93 a	65 a	212 a	0.9 a	0.0 a	0.3 ab	1.2 a	0.3 ab	0.0 a	0.3 a	0.2 ab	0.1 a	0.0 ab	3 b
	RedoxConN_2	36 ab	6 c	86 ab	61 a	199 ab	0.9 a	0.0 ab	0.3 a	1.1 ab	0.3 a	0.0 a	0.3 a	0.3 a	0.1 a	0.0 ab	1 b
	p	**0.0003**	**0.0004**	**0.0061**	**<0.0001**	**<0.0001**	**0.0040**	**0.0079**	**0.0002**	**0.0410**	**0.0292**	0.5280	0.0842	**0.0487**	0.7474	0.0642	**0.0156**
Accelerated aging	PD	26 ab	17 a	53 ab	47 a	141 b	0.8 c	0.0 a	0.1 a	0.6 a	0.1 a	0.0 a	0.4 a	0.1 a	0.0 a	0.1 a	10 a
	PO	28 ab	14 ab	66 a	47 a	147 b	0.8 c	0.0 a	0.2 a	0.8 a	0.2 a	0.0 a	0.4 a	0.1 a	0.1 a	0.1 a	6 abc
	AirMix	22 b	12 bc	48 ab	38 b	144 b	0.8 bc	0.0 a	0.2 a	0.8 a	0.2 a	0.0 a	0.4 a	0.2 a	0.1 a	0.1 a	9 ab
	N_2Mix	34 a	9 cd	57 ab	45 ab	168 ab	0.9 abc	0.0 a	0.2 a	0.6 a	0.2 a	0.0 a	0.2 b	0.3 a	0.1 a	0.1 a	4 bc
	RedoxConAir	25 ab	10 cd	60 ab	52 a	181 a	1 ab	0.0 a	0.2 a	0.7 a	0.2 a	0.0 a	0.2 b	0.1 a	0.1 a	0.1 a	3 c
	RedoxConN_2	20 b	7 d	44 b	48 a	190 a	1.1 a	0.0 a	0.2 a	0.6 a	0.2 a	0.0 a	0.2 b	0.1 a	0.0 a	0.0 a	2 c
	p	**0.0103**	**0.0001**	**0.0421**	**0.0045**	**0.0005**	**0.0040**	0.3853	**0.0005**	0.1602	0.2969	0.4123	**<0.0001**	0.6821	0.8668	0.1079	**0.0013**
Odor threshold (μg/L)		20 (I)	3 (I)	30 (II)	14 (I)	100 (III)	5 (I)	200 (I)	75 (IV)	250 (II)	1.1 (I)	30 (II)	50 (VI)	25 (I)	30 (V)	90 (VI)	0.09 (I)

*Different letters within a column for each sampling stage indicate significant differences for Fisher's LSD test and p < 0.05. Significant p-values are shown in bold fonts. Odor thresholds determined by Ferreira, 2000 (I); Guth, 1997 (II); Takeoka, 1993 (III); Poitou, 2021 (IV). Padrayuttawat, 1997 (V); Elsharif, 2016 (VI).

Table S4. Basic juice chemistry of Pinot noir grapes on the day of harvesting.

Total soluble solids (Brix)	pH	Titratable acidity (g/L tartaric acid)	Malic acid (g/L)	Yeast assimilable nitrogen (YAN)	Iron (mg/L)	Copper (mg/L)	Weight per berry (g)	Seeds per berry	Liquid: solid ratio by weight
26.1	3.37	6.9	1.96	160	0.3	0.4	1.2	1.4	2.2: 1

Table S5. One-way analyses of variance (ANOVA) of the composition of the cap after alcoholic fermentation of Pinot noir wines produced with selected cap management protocols. Values represent the mean of three tank replicates (n = 3).

Maceration treatment	Seeds (% weight/weight)	Whole berries (% weight/weight)
PD	14 b	6 b
PO	16 ab	10 ab
AirMix	17 ab	12 a
N_2Mix	19 a	11 ab
RedoxConAir	15 b	12 a
RedoxConN_2	17 ab	12 a
p	**0.006**	**0.022**

*Different letters within a column for each sampling stage indicate significant differences for Fisher's LSD test and $p<0.05$. Significant *p* values are shown in bold fonts.

Table S6. Retention times of selected anthocyanins and flavonols identified in Pinot noir wines by HPLC-DAD.

Category	Compound	Retention time (min)
Anthocyanins	Delphinidin-3-glucoside	3.7
	Petunidin-3-glucoside	6.2
	Peonidin-3-glucoside	7.8
	Malvidin-3-glucoside	8.8
	Vitisin B	9.9
	Vitisin A	10.5
	Polymeric Pigments	17.5
Flavonols	Myricetin-3-glucoside	7.2
	Quercetin-3-gluconoride	9.6
	Quercetin-glucoside	9.9
	Laricitrin-3-glucoside	10.5
	Kaempferol-3-glucoside	11.0
	Isorhamnetin-3-glucoside	12.1
	Syringetin-3-glucoside	12.3
	Quercetin	13.4
	Kaempferol	15.3
	Isorhamnetin	15.9
Grape reaction product	2-S-glutathionyl caftaric acid	1.8

**Table S7.** Retention times of selected monomeric flavan-3-ols, dimers, and trimers identified in Pinot noir wines by LCMS.		
Category	Compound	Retention time (min)
Monomeric Flavan-3-ols	(+)-Catechin	16.0
	(-)-Epicatechin	17.5
	(-)-Epicatechin gallate	19.5
	(-)-Epigallocatechin	15.4
Dimers	Catechin-catechin 1	1.9
	Catechin-catechin 2	3.7
	Catechin-catechin 3	15.2
	Catechin-catechin 4	16.4
	Catechin-catechin 5	19.6
	A type 1	9.1
	A type 2	15.4
	A type 3	17.4
	A type 4	19.7
	Catechin-catechin gallate	12.0
Trimers	Catechin-catechin-catechin 1	9.6
	Catechin-catechin-catechin 2	16.4
	Catechin-catechin-catechin 3	18.0
Sulfonated flavan-3-ols	Gallocatechin-SO3H	3.2
	Catechin-SO_3H 1	4.6
	Catechin-SO_3H 2	5.5
	Catechin-SO_3H 3	8.3
	Catechin-SO_3H 4	9.3
	Catechin-SO_3H 5	12.4
	Catechin-SO_3H 6	13.8
	Gallocatechin-gallocatechin-SO_3H 1	5.9
	Gallocatechin-gallocatechin-SO_3H 2	10.5
	Catechin-catechin-SO_3H 1	9.8
	Catechin-catechin-SO_3H 2	16.3
	Gallocatechin-catechin-SO_3H 1	6.0
	Gallocatechin-catechin-SO_3H 2	9.6
	Gallocatechin-catechin-SO_3H 3	13.7
	Gallocatechin-catechin-SO_3H 4	15.2

**Table S8**. Retention times, regression equations and r^2 values obtained for selected volatile aroma compounds as measured by GC/MS using pure standards (>95%).			
Compound	Retention time (min)	Regression equation	CF r^2
Ethyl butyrate	7.50	$y = 11.54 * x + 1.98 * 10^4$	0.9997
Ethyl isovalerate	8.25	$y = 24.74 * x + 1.04 * 10^5$	0.9998
Isoamyl acetate	9.49	$y = 29.39 * x + 0.01$	0.9996
Ethyl hexanoate	12.53	$y = 136.22 * x - 0.04$	0.9994
Hexyl acetate	13.61	$y = 106.61 * x - 0.01$	0.9991
Ethyl n-octanoate	17.00	$y = 311.17 * x - 0.12$	0.9855
Ethyl decanoate	21.24	$y = 7762.87 * x^{1.52}$	0.9998
methyl salycilate	25.46	$y = 12097.57 * x + 63.27 * x^2 + 2.25 * 10^4$	0.9989
2-phenylethyl acetate	26.47	$y = 8511.67 * x^2 + 123.10 * x + 0.002$	0.9984
Ethyl cinnamate	35.47	$y = 1294231.04 * x^8 + 224.38 * x + 7.38 * 10^4$	0.9996
Geraniol	25.96	$y = 1237.71 * x^2 + 15.83 * x + 4.19 * 10^4$	0.9999
Cis-rose oxide	15.43	$y = 136.05 * x + 1.30 * 10^4$	0.9998
Linalool	19.06	$y = 11.96 * x + 2.70 * 10^4$	0.9991
β-citronellol	24.97	$y = 3333.35 * x^2 + 19.89 * x + 1.81 * 10^4$	0.9993
Nerol	27.28	$y = 1032.91 * x^2 + 12.19 * x + 3.08 * 10^4$	0.9997
β-ionone	30.24	$y = 5320856.72 * x^2 + 110.18 * x + 0.001$	0.9996
Nerolidol (trans)	33.54	$y = 105588.65 * x^2 + 9.56 * x + 2.77 * 10^4$	0.9998

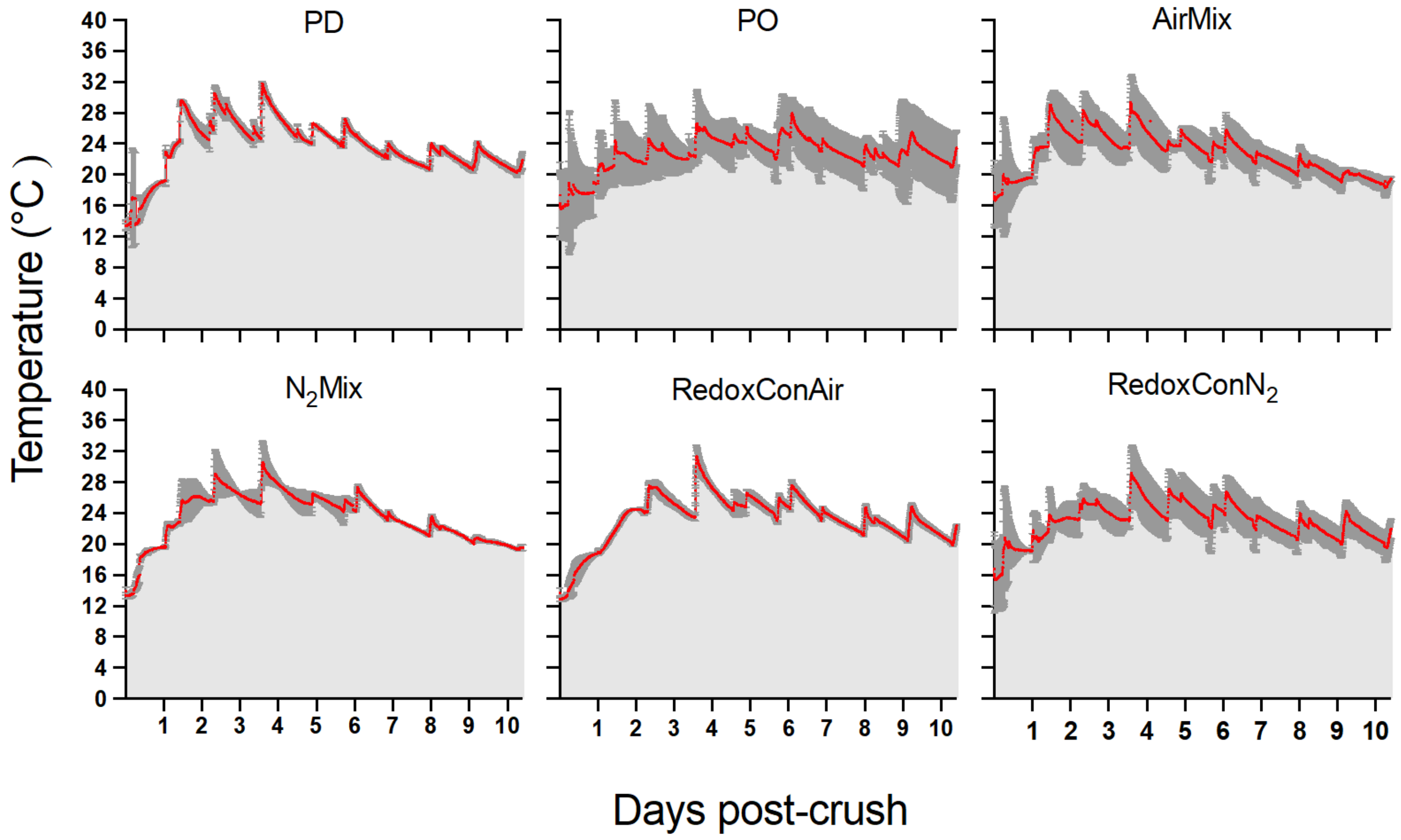

Figure S1. Temperature during alcoholic fermentation of Pinot noir wines made with varying cap management protocols. Each data point represents the mean of three replicates (n=3) with shading represents the standard deviation of these replicates

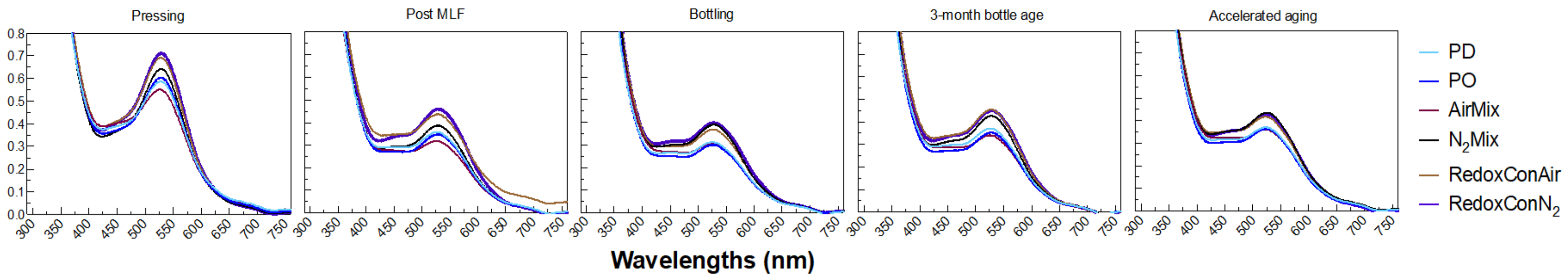

Figure S2. Full visible absorption spectrum scans of Pinot noir wines produced with selected cap management protocols, measured at selected time points of winemaking and aging. Data represents the mean of three replicates (n=3).

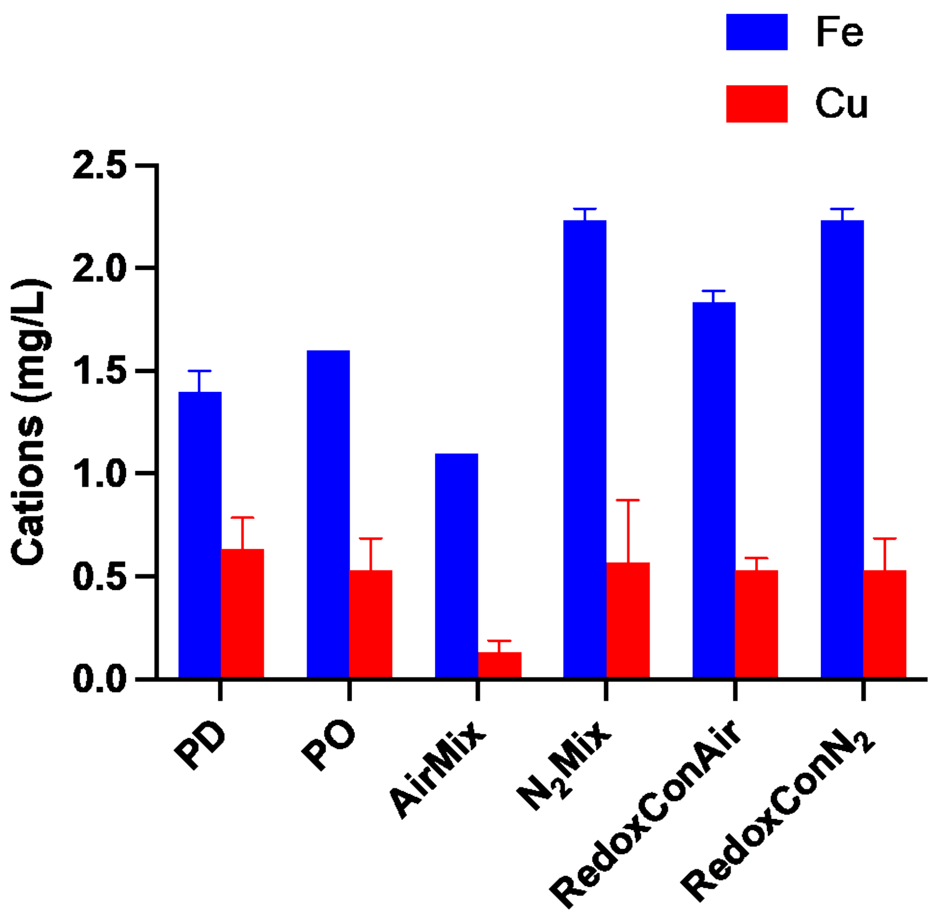

Figure S3. Iron (Fe) and copper (Cu) cation concentrations of Pinot noir wines produced with selected cap management protocols, measured at pressing. Data represents the mean of the replicates (n=3), and error bars represent the standard error
